# A Displaced Atypical Femoral Fracture Healed Without Anti-osteoporotic Agents in a Case of Ankylosing Spondylitis

**DOI:** 10.7759/cureus.70094

**Published:** 2024-09-24

**Authors:** Chang-Yu Huang, Chih-Yung Chiang, Kai-Chiang Yang, Chang-Chin Wu

**Affiliations:** 1 Department of Orthopedics, En Chu Kong Hospital, New Taipei City, TWN; 2 School of Dental Technology, College of Oral Medicine, Taipei Medical University, Taipei, TWN

**Keywords:** ankylosing spondylitis, atypical femoral fracture, bisphosphonate, osteoporosis, syndesmophyte

## Abstract

Bone loss leading to osteoporosis is a well-known feature in patients with ankylosing spondylitis (AS), with the prevalence of osteoporosis varying widely across different studies. However, there is still no consensus on the treatment of osteoporosis in AS patients. A 67-year-old male, a case of AS under medication control, had taken oral bisphosphonate for about seven years for suspected osteoporosis due to compression fracture at T12 and discontinued for disproportionately high dual-energy X-ray absorptiometry T-score (11.0 SD). He had been well until his right hip painful disability developed after a fall at home with a resultant right subtrochanteric transverse fracture with medial cortical spike, fulfilling features of atypical femoral fractures five months later. Open reduction and internal fixation with a cephalomedullary femoral nail were performed smoothly on the same day, and the fracture healed slowly and eventually one year later with only supplementation of calcium with vitamin D.

## Introduction

Bone loss leading to osteoporosis is a well-known feature in patients with ankylosing spondylitis (AS). The prevalence of osteoporosis in AS varies globally and has been reported between 19% and 62% in different studies [1,2]. The wide range of the prevalence may be related to the different measurements of the areal bone mineral density (aBMD). The aBMD at the lumbar spine may be overestimated due to the formation of syndesmophytes [3]. Despite the high prevalence of osteoporosis in AS patients, there is still no consensus on the treatment of osteoporosis in AS patients. Bisphosphonate is widely used to treat osteoporosis in the general population, but no sufficient data supports the use of bisphosphonate in AS patients. The evidence-based treatment is to control the disease activity with anti-tumor necrosis factor-alpha therapy [4]. There is a significantly increased BMD in the spine and hip in AS patients treated with infliximab. Besides, with an increase in patients receiving bisphosphonate for osteoporosis, adverse effects, including atypical femoral fracture (AFF), are gradually emphasized.

AFF is a rare type of fracture that usually occurs in the subtrochanteric region or the diaphysis of the femur [5]. The fracture is typically oriented perpendicular to the bone (transverse) or at a shallow angle (short oblique). Unlike typical osteoporotic fractures, AFFs usually do not have multiple bone fragments. A thickened cortex is often observed at the fracture site, especially on the lateral side of the femur, which can be visible under X-ray inspection before the fracture occurs.

Whether bisphosphonate treatment is beneficial for osteoporosis in AS patients remains unclear. This case presents a 67-year-old male with a medical history of AS and vertebral compression fracture who presented with a right femoral subtrochanteric fracture with AFF features, and the postoperative displaced fracture gap healed completely without the use of any anti-osteoporotic agent.

## Case presentation

A 67-year-old male had a medical history of hypertension and AS under medication control at rheumatologist’s clinics. The patient’s daily living activities score was normal, and he was able to walk unaided before the incident. He developed right hip painful disability after a fall in the bathroom. He was sent to our emergency room for evaluation. Plain X-ray films revealed a right subtrochanteric transverse fracture with medial cortical spike, transverse fracture line with medial oblique extension, lateral cortical breaking, and no comminution. These features met the criteria of AFF, and open reduction and internal fixation with cephalomedullary (CM) nailing using AO’s PFNA II implants (Depuy-Synthes, US) were performed on the same day.

**Figure 1:**
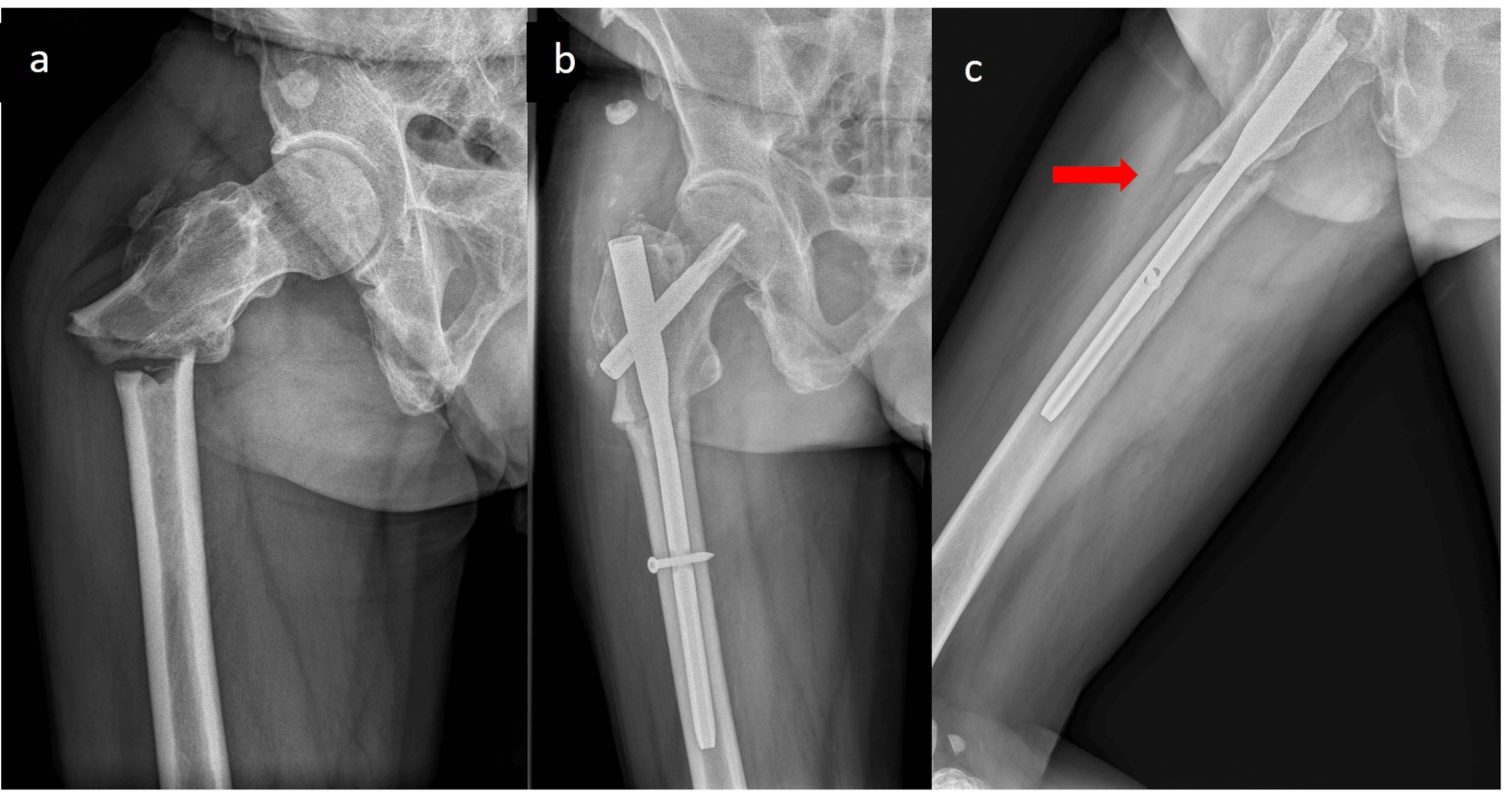
Pre and postoperative X-ray images of the fracture. (a) Right subtrochanteric transverse fracture with medial cortical spike, transverse fracture line with medial oblique extension, lateral cortical breaking, and no comminution meeting major features of atypical femoral fracture. (b) Mild varus displacement of the proximal femur. (c) A significant gap of about 0.5 cm in length revealed at the lateral view X-ray (red arrow).

The bone marrow canal reaming procedure had been challenging to execute for hard and condensed bone quality, and the perfect reduction had been jeopardized by the insertion of the nail, even with the smallest diameter. Before inserting the guide pin, we made a mini-open incision and used a Kocher clamp to reduce the proximal fragment. To avoid varus deformity, the entry point was placed slightly medial to the apex of the greater trochanter. A gap about 0.5 cm in length between fracture ends at the lateral view of postoperative X-ray and mild varus displacement of the proximal femoral fragment at the anteroposterior view was revealed (Figure 1).

**Figure 2:**
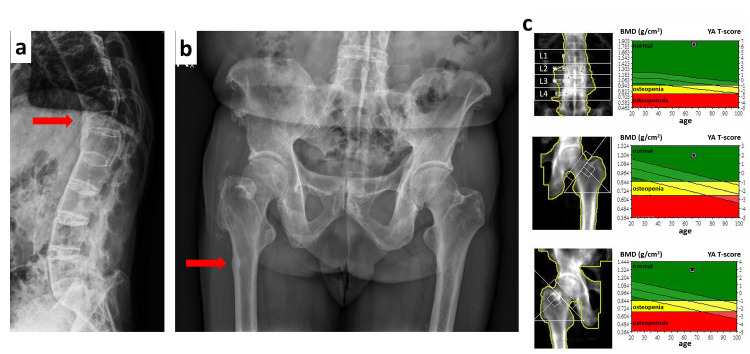
Spinal lateral view presented a typical bamboo spine, pelvis X-ray image, and dual-energy X-ray absorptiometry findings. (a) Spinal lateral view shows a typical bamboo spine and a collapsed T12 compression fracture with syndesmophyte formation. (b) Pelvis X-ray of the lateral femoral cortical bone just near the fracture site. (c) High bone marrow density in all three areas revealed by dual-energy X-ray absorptiometry.

Tracing back his medical history, AS was diagnosed when he was 30 years old due to low back pain. Initially, he had taken Chinese traditional medicine for disease control since AS was diagnosed. Severe kyphosis and back stiffness had developed about 10 years ago. He came to our rheumatologist’s outpatient clinic about eight years before the index surgery for severe back pain, while the X-ray films of the lumbar spine revealed a bamboo spine and a compression fracture at the T12 vertebral body (Figure 2a). Due to the compression fracture, osteoporosis was highly suspected, FOSAMAX PLUS® 70/5600 (alendronate sodium 70 mg/cholecalciferol 5,600 IU, MSD, US) had been prescribed for treatment despite no confirmation of osteoporosis by dual-energy X-ray absorptiometry (DXA). He also started to receive Sulfazine (sulfasalazine 500 mg QD) for disease control, while no steroids were used. Subsequently, the bisphosphonate was continuously dispensed for the treatment of osteoporosis without re-evaluations or drug holidays for more than seven years, and a total of 380 pills of FOSAMAX PLUS® were prescribed. About five months before the fracture occurred, he visited the rheumatologist’s clinic with a chief complaint of right thigh pain and underwent studies, including the pelvis X-ray revealing localized periosteal reaction of the lateral femoral cortical bone just near the fracture site (Figure 2b). The DXA study showed the T-scores were 1.9 standard deviations (SD) and 2.9 SD in the right hip and left hip, respectively. The spine T-scores of BMD were disproportionately high (11.0 SD) (Figure 2c).

**Figure 3:**
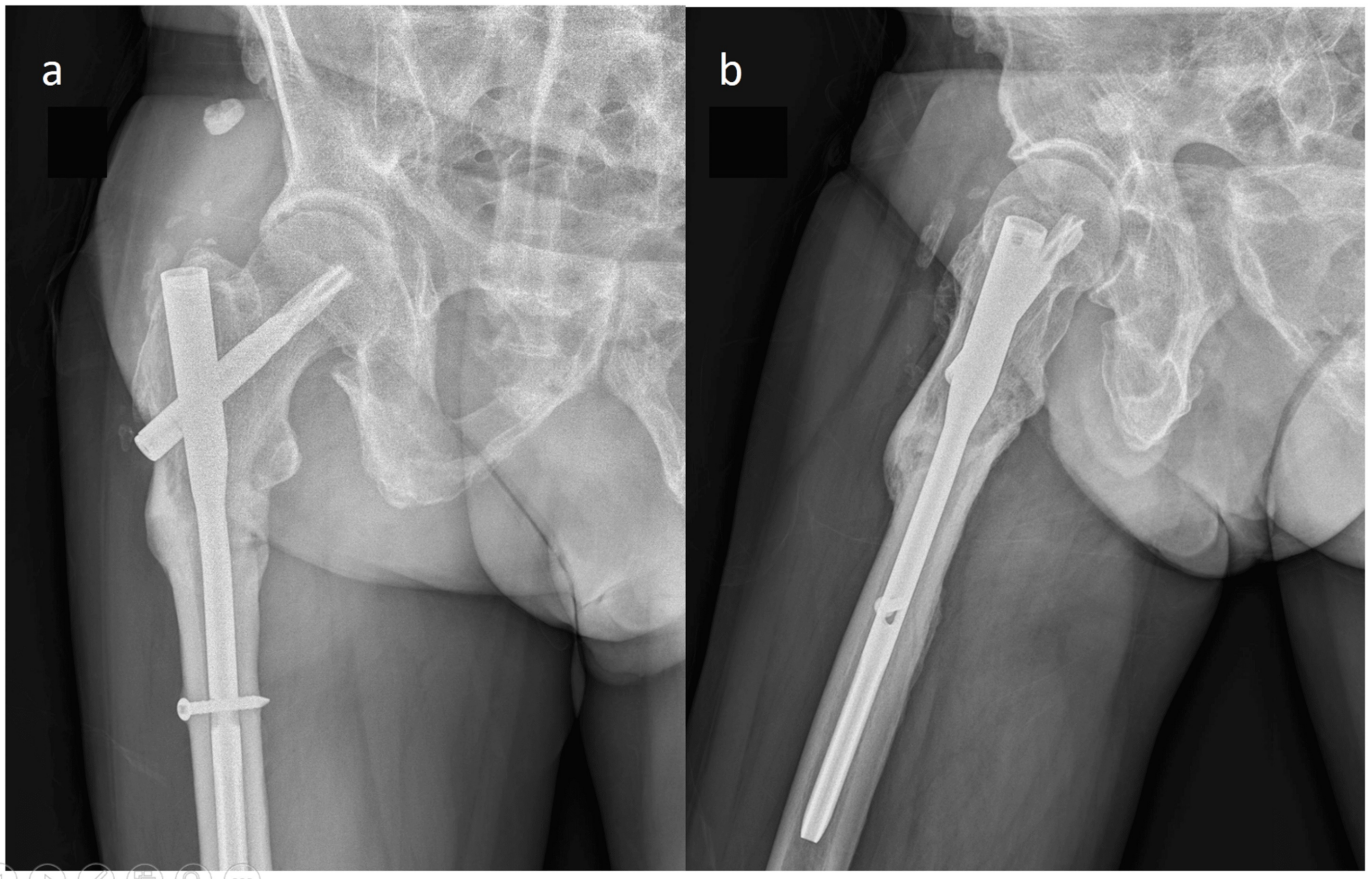
Bony union was achieved at one year. Bony union was achieved at the one-year follow-up X-ray, and the original gap was filled with newly formed bone.

Oral bisphosphonate was discontinued at that time due to relatively high BMD. The postoperative follow-up image revealed stable fixation, and the complete bony union was achieved at the one-year follow-up (Figure 3).

**Figure 4:**
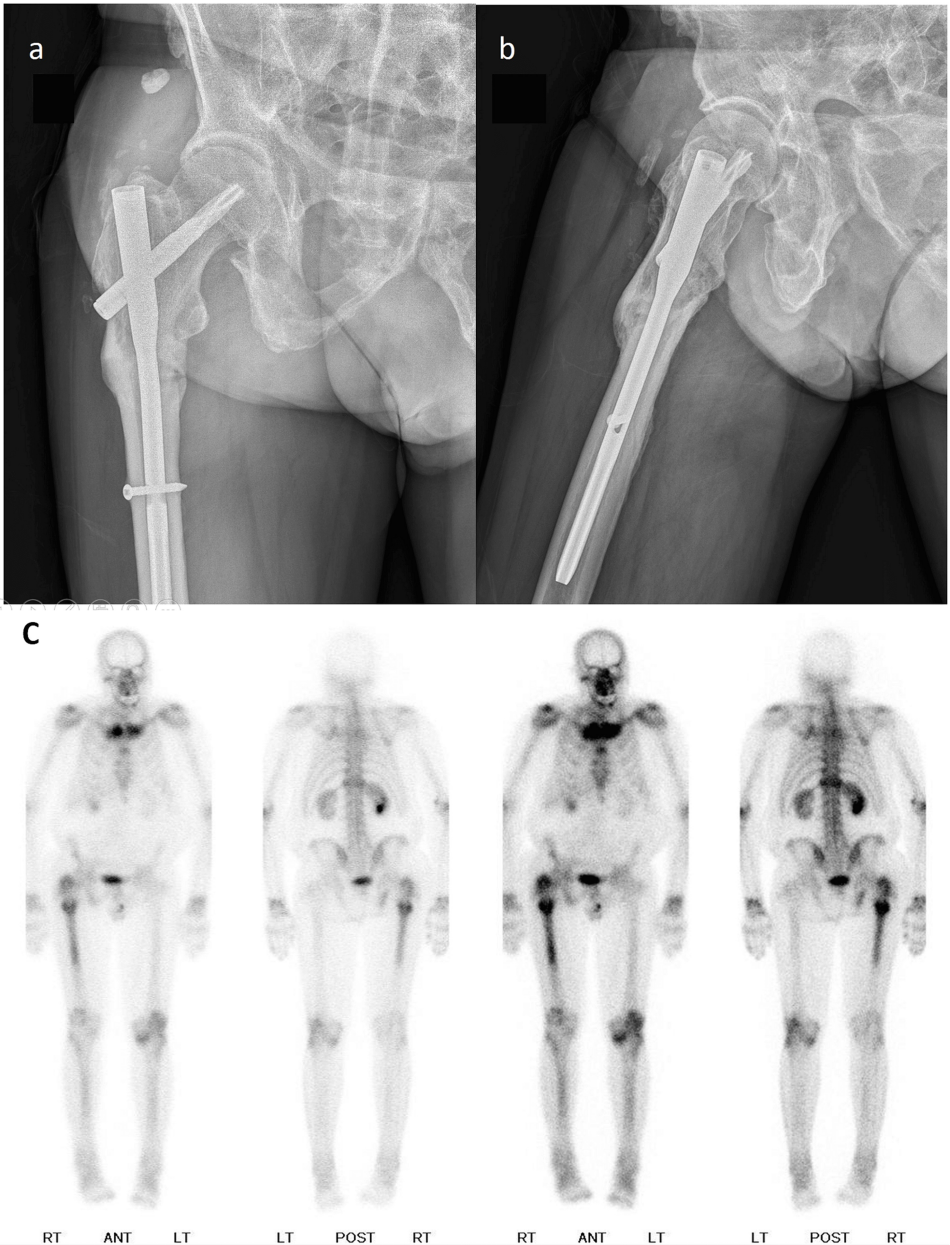
No atypical femoral fracture features were identified, and the whole-body scan. (a, b) No atypical femoral fracture features were identified on X-ray films of the left thigh. (c) The whole-body bone scan revealed no atypical femoral fracture hotspot.

He discarded the walker two weeks postoperatively and ambulated independently, but had complained of intractable right thigh soreness for the initial three months after the operation and had been relieved by celecoxib 200 mg QD and acetaminophen 500 mg Q6H. Right thigh pain subsided gradually and disappeared completely about six months later. No anabolic osteoporotic medications or other adjuvant therapies, such as low-intensity pulsed ultrasound, had been dispensed despite the diagnosis of AFF, with only supplementation of calcium and vitamin D dispensed (tribasic calcium phosphate 1,203 mg and cholecalciferol 330 IU BIO-CAL PLUS 1 # TID) till complete union. Left femoral X-ray and bone scan studies were performed despite no left hip pain or left thigh pain complaint after four years of follow-up to find contralateral insufficient AFF. The plain X-ray revealed no typical AFF features at the left femur, and the bone scan showed no hotspot at the lateral cortex (Figure 4).

**Figure 5:**
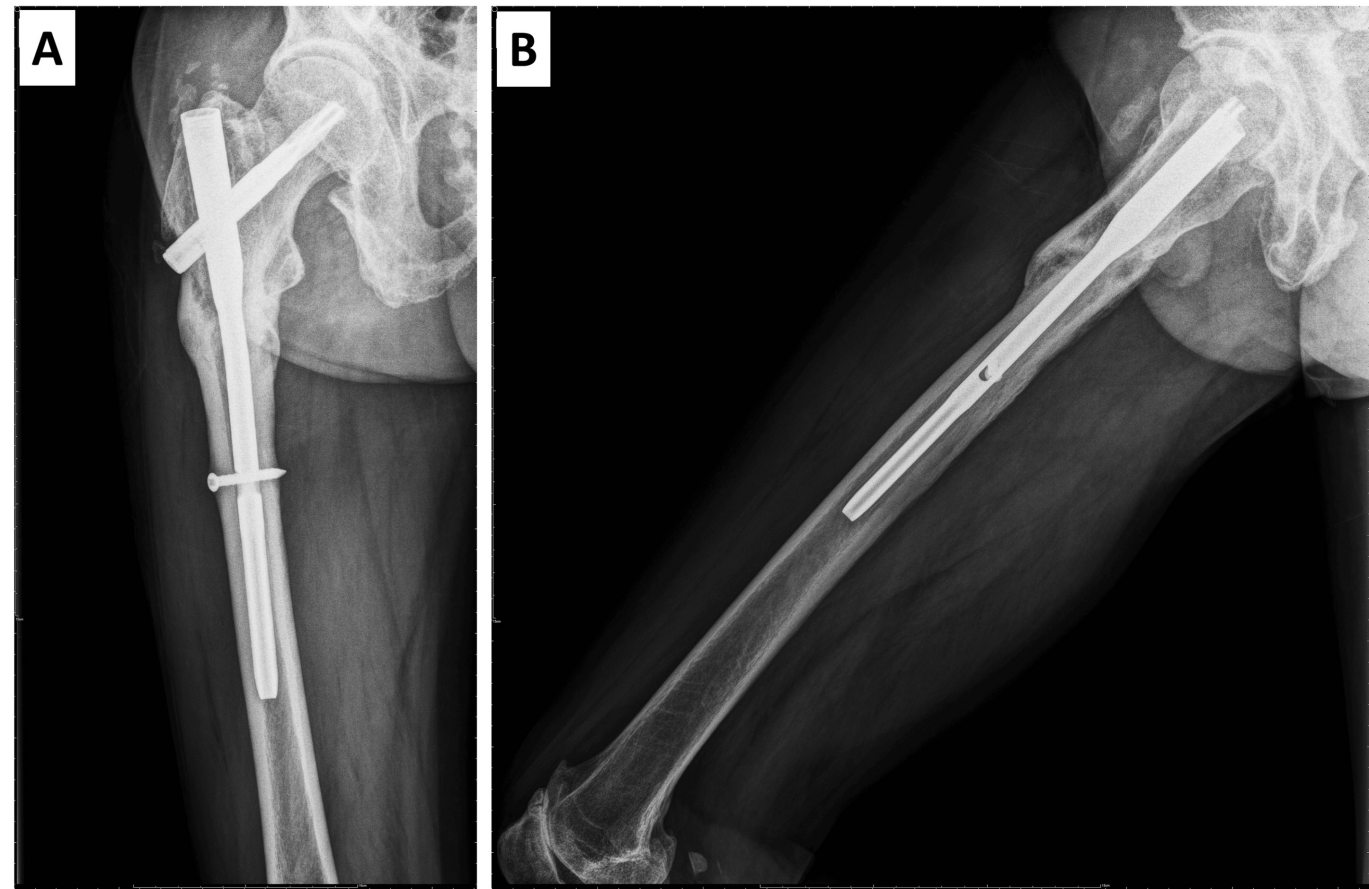
X-ray taken two years after surgery. X-ray taken two years after surgery revealing a solid union at the previous fracture site.

The X-ray taken two years after surgery revealed a solid union at the previous fracture site (Figure 5).

## Discussion

This case of AS encountered an AFF after bisphosphonate treatment for a thoracic compression fracture caused by possible osteoporosis for more than seven years. The association between this AFF and bisphosphonate treatment was obvious. Bisphosphonate therapy appears to increase the risk of atypical fracture, with greater risk associated with a longer duration of treatment and declining risk after discontinuation of bisphosphonate [6].

In 2014, the task force report from the American Society for Bone and Mineral Research reviewed the clinical data published and suggested the following treatment recommendations for patients with AFFs: (1) discontinue bisphosphonate therapy once the diagnosis of AFFs is established and reassess the initiation of calcium and vitamin D treatment; (2) conduct prophylactic intramedullary nailing operations for patients with incomplete fractures who are suffering from pain [7].

Femoral subtrochanteric and shaft fractures are usually treated with intramedullary (IM) nailing or plating. The preference toward IM nailing is explained by the fact that bone healing through endochondral ossification is usually not achievable with a plate. IM nails are load-sharing devices that are stiffer than plates. The fracture is not exposed during nailing, and the periosteum is not interrupted. Therefore, the blood supply is preserved to facilitate revascularization following bone healing. However, Weil et al. reported that 54% (17 AFF cases) of the IM nailing-treated patients could achieve normal healing, and 46% needed the secondary procedure [8]. In the author’s opinion, a qualitative bone defect caused by prolonged bisphosphonate therapy could be a causative mechanism of delay union or non-union. Furthermore, drugs impairing osteoclast function or reducing bone remodeling might delay or impair bone healing [9]. In a human bone biopsy study on long-term bisphosphonate-treated patients presenting with cortical fractures, suppressed bone turnover was found in the vast majority (10 of 15) [10]. For suppressed bone metabolism, teriparatide may be a choice of treatment. Therefore, treatment of AFF is currently challenging. CM nailing was applied in this case to guarantee early weight-bearing of the affected side and protect against possible insufficient AFF of the contralateral side, as suggested by Javaid et al. that CM nailing indicated for initial encouragement of AFF to protect the contralateral side [11]. No anabolic anti-osteoporotic agents were dispensed for this AS case due to no available reference revealing the benefits of anabolic agents in AS patients with AFF and rapid fracture healing and bony ingrowth. He was only prescribed oral calcium and vitamin D supplementation and could ambulate independently with no assistive device for two weeks. After 12 months, the fracture healed completely and was eventually revealed by follow-up X-rays with no additional procedure despite the malalignment and reduction gap mentioned before. The acceptable results were similar to those of Egol et al., who surgically treated bisphosphonate-associated complete AFF fixed by IM nailing and achieved generally reliable results, although delayed fracture healing, if malaligned, and nearly two-thirds returned to self-reported baseline function within one year [12].

Another concern in this case was whether bisphosphonate treatment for AS patients is necessary. No evidenced result supports bisphosphonate treatment in AS patients. However, bisphosphonate was recommended in AS patients with osteoporosis by experts involved in the 3E Initiative in Rheumatology [13]. Bisphosphonate treatment should be considered in patients older than 50 years of age with hip or vertebral fracture and T-score ≤-2.5 SD at the femoral neck. In our case, there was no BMD survey before bisphosphonate treatment. Re-evaluation of the necessity of continuity of bisphosphonate is required at three years or five years for prevention of adverse effects. The other important factor influencing treatment strategy is the BMD result. DXA is the most commonly used method to detect osteoporosis in the general population. However, various studies have pointed out that the spinal BMD measured through DXA may not correctly reflect the osteoporotic fragility. The syndesmophyte formation may mask bone loss in the lumbar spine, and a false increase may be measured in the lumbar BMD values. In this case, the spine BMD was disproportionately high compared with the value in the hip. We might make a mistaken diagnosis of non-osteoporotic status in AS patients according to spine BMD value. BMD in the femoral neck is more valuable in AS patients. The BMD T-scores of hips, in this case, were 2.9 SD and 1.9, which supported us in discontinuing the bisphosphonate treatment for vertebral compression fracture and suspected osteoporosis. However, the correlation between the T-score and the risk of AFF has not been established. Some studies focused on T-score discordance of BMD in AFF patients. Lee et al. found that the prevalence of T-score discordance between the hip and lumbar spine was relatively high in patients with AFF [14]. In our case, the T-score discordance was significant.

Patients with AS, especially in advanced stages, are at a higher risk of osteoporosis and bone weakening, which increases their risk of fractures. Bisphosphonates, by increasing bone density, could theoretically reduce this risk. While bisphosphonates are effective in reducing fracture risk in osteoporotic patients, the evidence for their role in preventing fractures, specifically in AS patients, is limited. Some studies suggest that they can help increase BMD in AS, which may contribute to fracture prevention. AS patients are particularly prone to spinal fractures due to the rigid, fused nature of their spines. However, the effectiveness of bisphosphonates in preventing such fractures remains unclear, as these fractures are often more related to the structural changes of AS than to bone density alone. Therefore, additional case studies are needed for the management of bisphosphonate-induced AFF in AS patients with relatively high BMD.

Bisphosphonates, commonly used to treat osteoporosis, have shown some potential benefits for patients with AS. First, AS can lead to osteoporosis or low bone density, particularly in advanced cases. Bisphosphonates, such as alendronate, have been studied for their ability to improve BMD in AS patients. Second, some studies suggest that bisphosphonates may reduce inflammation and provide pain relief in AS patients due to their anti-inflammatory properties [15]. However, this effect is generally mild compared to traditional anti-inflammatory treatments such as non-steroidal anti-inflammatory drugs or biologics. Third, limited evidence suggests that bisphosphonates could slow the progression of spinal fusion in AS. However, long-term use of bisphosphonates in a patient (more than seven years in this patient) with AS raises several potential concerns, including the risk of AFFs, osteonecrosis of the jaw, suppression of normal bone turnover, and the potential for reduced effectiveness over time.

## Conclusions

In this case, surgical reduction with CM nailing fixation and discontinuation of bisphosphonate with only calcium and vitamin D supplementation could meet optimal clinical results and complete bony union despite a significant gap and mild alignment. Calcium and vitamin D play important roles in fracture healing and bone remodeling. Calcium and vitamin D deficiencies may negatively influence fracture healing. Many animal studies have supported the benefits of calcium and vitamin D supplementation in fracture healing. However, clinical studies addressing the effects of calcium and vitamin D supplementation on fracture healing are rare, and the results remain inconclusive. Calcium and vitamin D are relatively inexpensive products and rarely cause adverse effects. In brief, we think calcium and vitamin D supplementation is very safe and may benefit fracture healing and remodeling without negative effects.
